# PDBeCIF: an open-source mmCIF/CIF parsing and processing package

**DOI:** 10.1186/s12859-021-04271-9

**Published:** 2021-07-23

**Authors:** Glen van Ginkel, Lukáš Pravda, José M. Dana, Mihaly Varadi, Peter Keller, Stephen Anyango, Sameer Velankar

**Affiliations:** 1 European Molecular Biology Laboratory, European Bioinformatics Institute (EMBL-EBI), Wellcome Genome Campus, Hinxton, UK; 2 Global Phasing Ltd., Sheraton House, Castle Park, Cambridge, CB3 0AX UK

**Keywords:** Software, Parser, PDB, PDBx/mmCIF, Protein structure, CCD, Small molecule

## Abstract

**Background:**

Biomacromolecular structural data outgrew the legacy Protein Data Bank (PDB) format which the scientific community relied on for decades, yet the use of its successor PDBx/Macromolecular Crystallographic Information File format (PDBx/mmCIF) is still not widespread. Perhaps one of the reasons is the availability of easy to use tools that only support the legacy format, but also the inherent difficulties of processing mmCIF files correctly, given the number of edge cases that make efficient parsing problematic. Nevertheless, to fully exploit macromolecular structure data and their associated annotations such as multiscale structures from integrative/hybrid methods or large macromolecular complexes determined using traditional methods, it is necessary to fully adopt the new format as soon as possible.

**Results:**

To this end, we developed PDBeCIF, an open-source Python project for manipulating mmCIF and CIF files. It is part of the official list of mmCIF parsers recorded by the wwPDB and is heavily employed in the processes of the Protein Data Bank in Europe. The package is freely available both from the PyPI repository (http://pypi.org/project/pdbecif) and from GitHub (https://github.com/pdbeurope/pdbecif) along with rich documentation and many ready-to-use examples.

**Conclusions:**

PDBeCIF is an efficient and lightweight Python 2.6+/3+ package with no external dependencies. It can be readily integrated with 3rd party libraries as well as adopted for broad scientific analyses.

**Supplementary Information:**

The online version contains supplementary material available at 10.1186/s12859-021-04271-9.

## Background

The Worldwide Protein Data Bank (wwPDB) [1] organization manages the Protein Data Bank Archive (PDB) [2]—the single global archive of experimentally determined 3D-structure data. The consortium members - Research Collaboratory for Structural Bioinformatics Protein Data Bank (RCSB PDB) [3], the Protein Data Bank in Europe (PDBe) [4], Protein Data Bank Japan (PDBj) [5] and BioMagResBank (BMRB) [6] collaborate on deposition, validation, biocuration, and open access dissemination of macromolecular 3D-structure data.

Since 1970, the PDB structures have been distributed using the legacy, human-readable PDB file format. However, rapid advances in experimental and structure determination methods such as cryo-electron microscopy and integrative/hybrid methods quickly revealed its limitations [2, 7]. The new standard of the Protein Data Bank, the PDBx/mmCIF became the master format for the PDB archive in 2014 [8]. The new format utilizes printable characters from the ASCII set and is based on a data exchange dictionary that is maintained and further expanded by the wwPDB consortium. Its thorough description is available at https://mmcif.wwpdb.org/**.** Briefly, it is an extension of the CIF format [9], the gold standard in small molecule crystallography. Each file contains one or more data blocks pre-fixed with ‘data_’ and populated with data items. Each data item is uniquely identified by a leading underscore and a name. The name is composed of two parts separated by a period: category and keyword. There are two types of categories: key-value and tabular. Key-value features just a single value of type string per keyword, while tabular is an array of strings. Since the CIF format is derived from the syntax of the Self-defining Text Archive and Retrieval (STAR) [10] format, PDBeCIF relies on a community established solution for tokenization (startools [11]**)** to aid file interpretation.

The PDBx/mmCIF format superseded the PDB file format to remove size restrictions on deposited structures and to greatly improve the representation of additional information distributed alongside the coordinates. Indeed, the legacy format supported additional information only to some extent using REMARK fields; e.g. REMARK 350 presents information about crystallographic and non-crystallographic transformations to create biologically functional biomolecules. Nevertheless, this information used to be stored as free text and hence cumbersome to access programmatically. On the other hand, PDBx/mmCIF files contain programmatically accessible information about structural elements of the macromolecular assemblies (category: pdbx_struct_assembly), details on the generation of such assemblies (pdbx_struct_assembly_gen), their properties and features (pdbx_struct_assembly_prop) and much more. Such level of clarity is achieved by employing PDBx/mmCIF Exchange Dictionary that defines the validation of data item values using data types, controlled dictionaries and ranges. The introduction of a controlled dictionary adheres to FAIR principles [12] (i.e. Findable, Accessible, Interoperable and Reusable). For example the data item ‘refine.pdbx_method_to_determine_struct’ allows 9 different values for new depositions, which is in direct contrast with dozens of distinct data item values used in previous years.

Despite these improvements, the adoption of this new file format by the scientific community is still ongoing. Indeed, some of the popular software tools still rely on the legacy PDB format [13–16], and even newly developed software may lack support for the mmCIF format [17, 18]. To ensure backwards compatibility with scientific software for the foreseeable future, the wwPDB provides structures in the legacy PDB format for a subset of a PDB format compliant archive. Nevertheless, the overall direction is to develop PDBx/mmCIF to represent rich metadata and associated annotations as evident by the recent decision to make the Macromolecular Crystallographic Information File format (PDBx/mmCIF) mandatory for crystallographic depositions of new structures even when these are compliant with the legacy format [7]. The anticipated extension of the PDB small molecule identifiers defined by the Chemical Component Dictionary beyond the limits of the legacy PDB format will result in their general incompatibility in the near future.

The community would, therefore, benefit from an accelerated adoption rate of the new data standard by making more software PDBx/mmCIF format compliant. To facilitate this transition, we present a lightweight, general-purpose Python package, PDBeCIF. This package allows reading from and saving to PDBx/mmCIF files, reading Crystallographic Information Files (CIF), and provides several convenient methods for optimized search of the file content.

## Implementation

The PDBeCIF package can be readily installed from PyPI or GitHub and has no external dependencies. Both Python 3 and legacy Python 2 are supported. Rich documentation with many use case examples is available at https://pdbeurope.github.io/pdbecif/ along with on-demand online training explaining its functionality in detail https://pdbeurope.github.io/api-webinars/webinars/web5/pdbecif.html.

The package contains several classes. Two of them allow reading (CifFileReader) and writing (CifFileWriter) of PDBx/mmCIF files. The output of the file reading can be either a plain Python dictionary, an object representation available as a CIFWrapper object or a CifFile object. Each of the objects covers a slightly different use case (see Additional file 1) for details.

**Fig. 1:**
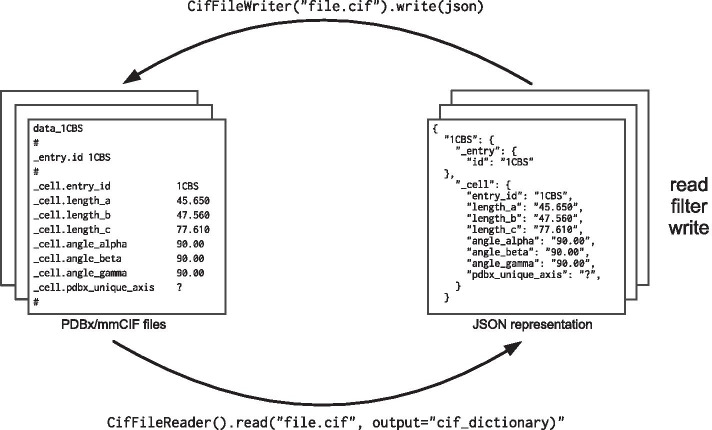
Schema of PDBx/mmCIF file reading/writing using PDBeCIF package. The example shows code snippets and the result as a Python dictionary corresponding to an updated PDB entry “1cbs” section.

First, the input file can be written into a hierarchy of plain Python dictionaries. The key at the first level is equal to the data block id and the value that corresponds to this key is another dictionary with category names as keys (Fig. 1).

Second, CIFWrapper is a wrapper object that allows accessing the content of the file with Python dot notation and exposes search functions to filter data items based on string conditions and regular expressions.

Finally, the CIFFile data object allows easy modification of the content of an mmCIF file, including the addition and removal of categories and data items. The parser contains a mechanism that allows discarding unwanted categories and the extraction of selected categories, further improving parsing speed and memory efficiency. Export of PDBx/mmCIF files can be done using CifFileWriter and its write method that accepts all above-mentioned objects as a parameter.

## Results

As described above, one of the advantages of the PDBx/mmCIF file format is the inclusion of additional information alongside the coordinates making the data compliant with the FAIR principles and providing a more complete biological context. In many cases, this information is fragmented and can only be obtained by a combination of different specialist resources e.g. [3, 19, 20]**.** PDBe makes updated PDBx/mmCIF files available that feature additional information. These files promote consistent and standardized metadata on the top of the core PDB archive information, facilitating further expansion of the core Exchange Dictionary. The recently established, community-driven PDBe-KB resource [21] collates the biological context of the coordinates and may further require expansion of the Exchange Dictionary to support an increased number of annotations being distributed as part of PDB mmCIF files. For example, connectivity information for all the building blocks in a PDB entry is encoded in the category ‘_chem_comp_bond’. Ligands and other small molecules listed in the Chemical Component Dictionary (CCD) come with additional information that could be extracted too, e.g. information about mapping to other small molecule databases, common synonyms, or DrugBank [22] classifications to name a few.

**Fig. 2:**
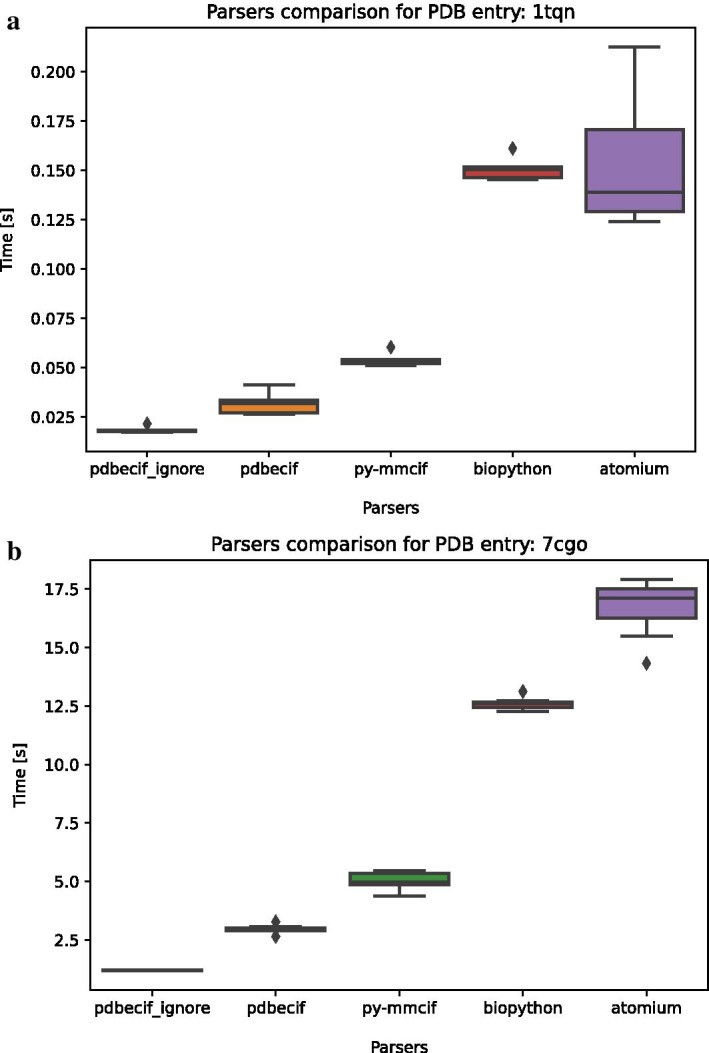
Comparative analysis of mmCIF parsers available from Python. PDBeCIF package was the fastest in both cases with parsing times of 0.3 s, and 2.28 s respectively. We achieved further speed optimization by discarding the *atom_site* category (pdbecif_ignore column). One reason for atomium or Biopython being considerably slower than PDBeCIF is that PDBeCIF is a pure algorithmic parser that does not make any structure interpretation. **a** Comparison of parsing speed for small protein (PDB id: 1tqn, 3999 atoms). **b** Comparison of parsing speed for a sizeable flagellar motor-hook (PDB id: 7cgo; ~ 335K atoms).

We carried out a comparative performance analysis between PDBeCIF v1.5 and some of the other popular mmCIF parsers available in Python such as Biopython v1.78 [23] and py-mmcif v0.67 [24]. We also selected a representative of a current mmCIF parser—atomium v1.0.9 [25]. We carried out the benchmarking on a MacBook Pro (2.9 GHz Quad-Core Intel Core i7) in python 3.9.2 installed using the conda package manager. We measured the running time on 7 consecutive runs and averaged the values. We choose a small protein (PDB id: 1tqn) and a large molecular machine (PDB id: 7cgo) for comparisons. Results of this analysis are available in Fig. 2.

In conclusion, PDBeCIF is a lightweight Python 2/3 package with no dependencies, that allows manipulating mmCIF/CIF files distributed by the wwPDB partners. The project is open source, maintained by the PDBe team and employed in the PDBe production processes which ensure its continued development and maintenance. It can be readily integrated into any Python project or used on the interface between software modules for format conversion, hopefully facilitating the more widespread adoption of the PDBx/mmCIF format.

## Supplementary Information

**Additional file 1**. Use cases and code examples.

## Data Availability

Project name: PDBeCIF. Project home page: https://github.com/pdbeurope/pdbecif. Operating system(s): Platform independent. Programming language: Python. Other requirements: Python 2.6+/3+. License: Apache 2.0. Any restrictions to use by non-academics: see the license. PDBeCIF is freely available at PyPI repository (http://pypi.org/project/pdbecif) and from GitHub (https://github.com/pdbeurope/pdbecif) along with rich documentation and many ready-to-use example.

## Abbreviations

wwPDB: Worldwide Protein Data Bank
PDBx/mmCIF: macromolecular crystallographic information file format
CIF: crystallographic information files
ASCII: American Standard Code for Information Interchange
STAR: self-defining text archive and retrieval
CCD: chemical component dictionary
