# Supplementary material for: PDBeCIF: an open-source mmCIF/CIF parsing and processing package
Source: BMC Bioinformatics. 2021 Jul 23;22:383. doi: 10.1186/s12859-021-04271-9 (PMC8299628; doi:10.1186/s12859-021-04271-9)
Supplement: Supplementary file 1 — Additional file 1. Use cases and code examples. [file 12859_2021_4271_MOESM1_ESM.docx]

PDBeCIF: an open-source mmCIF/CIF parsing and processing package

Glen Van Ginkel^1^, Lukáš Pravda^1^, José M Dana^1^, Mihaly Varadi^1^, Peter Keller^2^, Stephen Anyango^1^, Sameer Velankar^1^

^1^ European Molecular Biology Laboratory, European Bioinformatics Institute (EMBL-EBI), Wellcome Genome Campus, Hinxton, UK

^2^ Global Phasing Ltd., Sheraton House, Castle Park, Cambridge CB3 0AX, UK

Additional information

## Use case 1

Example use of cif_dictionary mode to extract DrugBank targets for Imatinib (STI) <http://ftp.ebi.ac.uk/pub/databases/msd/pdbechem_v2/S/STI/STI.cif>. The dictionary output is useful when no filtering is required and a plain Python dictionary object is sufficient.

from pdbecif.mmcif_io import CifFileReader

reader = CifFileReader()

cif_dictionary = reader.read("STI.cif", output="cif_dictionary")

# traverse the dictionary hierarchy to obtain UniProt ids of known targets.

STI_data_block = cif_dictionary['STI']

drugbank_targets_category = STI_data_block['_pdbe_chem_comp_drugbank_targets']

known_targets = drugbank_targets_category['uniprot_id']

## Use case 2

Use of cif_wrapper mode to extract connectivity information from an updated PDB entry for Retinoic acid (REA). This example is using an updated entry of 1cbs for this demonstration. The file is available from <https://www.ebi.ac.uk/pdbe/entry-files/download/1cbs_updated.cif>. The cif wrapper is useful for searching in the mmCIF categories.

from pdbecif.mmcif_io import CifFileReader

reader = CifFileReader()

cif_wrapper = reader.read("1cbs_updated.cif", output="cif_wrapper", only="_chem_comp_bond")

# extract the first data block

data_block = list(cif_wrapper.values())[0]

# search for bonds within retinoic acid returns Python dictionary

rea_bonds = data_block._chem_comp_bond.search("comp_id", "REA")

for identifier, bond in rea_bonds.items():

print(f"{bond['atom_id_1']} - {bond['atom_id_2']} {bond['value_order']}")

## Use case 3

Use cif_file mode to modify the content of the mmCIF file and store the result in another mmCIF file. This example uses an updated mmCIF file for PDB entry 5hht for this demonstration. The file is available from <https://www.ebi.ac.uk/pdbe/entry-files/download/5hht_updated.cif> and REST API endpoint that fetches information about cofactor molecules present in the PDB entry (<https://www.ebi.ac.uk/pdbe/api/pdb/entry/cofactor/5hht>).

from pdbecif.mmcif_io import CifFileReader, CifFileWriter

import requests

reader = CifFileReader()

writer = CifFileWriter('new_cif_file.cif')

cif_file = reader.read("5hht_updated.cif", output="cif_file")

# extract the first data block

data_block = cif_file.getDataBlocks()[0]

# Retrieve cofactor data from external API and format them into data items

# The idea is to have dictionary with key equal to mmCIF keyword and value

# is the list of values for a given keyword

response = requests.get('https://www.ebi.ac.uk/pdbe/api/pdb/entry/cofactor/5hht')

data = response.json()

cofactor_info = data['5hht']

results = {}

for entry in cofactor_info:

for key, value in entry.items():

if key in results:

results[key].append(value)

else:

results[key] = [value]

print(results)

# add the new category

new_category = data_block.setCategory("cofactor_information")

for key, value in results.items():

new_item = new_category.setItem(key)

new_item.setValue(value)

writer.write(cif_file)
